# CTGF in kidney fibrosis and glomerulonephritis

**DOI:** 10.1186/s41232-018-0070-0

**Published:** 2018-08-06

**Authors:** Naohiro Toda, Masashi Mukoyama, Motoko Yanagita, Hideki Yokoi

**Affiliations:** 1 Department of Nephrology, Graduate School of Medicine, Kyoto University, 54 Shogoin Kawahara-cho, Sakyo-ku, Kyoto, 606-8507 Japan; 2 Department of Nephrology, Kumamoto University Graduate School of Medical Sciences, Kumamoto, Japan

**Keywords:** CTGF, Glomerulonephritis, Inflammation, Macrophage, Chemokine

## Abstract

**Background:**

Glomerulonephritis, which causes inflammation in glomeruli, is a common cause of end-stage renal failure. Severe and prolonged inflammation can damage glomeruli and lead to kidney fibrosis. Connective tissue growth factor (CTGF) is a member of the CCN matricellular protein family, consisting of four domains, that regulates the signaling of other growth factors and promotes kidney fibrosis.

**Main body of the abstract:**

CTGF can simultaneously interact with several factors with its four domains. The microenvironment differs depending on the types of cells and tissues and differentiation stages of these cells. The diverse biological actions of CTGF on various types of cells and tissues depend on this difference in microenvironment. In the kidney, CTGF is expressed at low levels in normal condition and its expression is upregulated by kidney fibrosis. CTGF expression is known to be upregulated in the extra-capillary and mesangial lesions of glomerulonephritis in human kidney biopsy samples. In addition to involvement in fibrosis, CTGF modulates the expression of inflammatory mediators, including cytokines and chemokines, through distinct signaling pathways, in various cell systems. In anti-glomerular basement membrane (GBM) glomerulonephritis, systemic CTGF knockout (Rosa-CTGF cKO) mice exhibit 50% reduction of proteinuria and decreased crescent formation and mesangial expansion compared with control mice. In addition to fibrotic markers, the glomerular mRNA expression of *Ccl2* is increased in the control mice with anti-GBM glomerulonephritis, and this increase is reduced in Rosa-CTGF cKO mice with nephritis. Accumulation of MAC2-positive cells in glomeruli is also reduced in Rosa-CTGF cKO mice. These results suggest that CTGF may be required for the upregulation of *Ccl2* expression not only in anti-GBM glomerulonephritis but also in other types of glomerulonephritis, such as IgA nephropathy; CTGF expression and accumulation of macrophages in the mesangial area have been documented in these glomerular diseases. CTGF induces the expression of inflammatory mediators and promotes cell adhesion.

**Short conclusion:**

CTGF plays an important role in the development of glomerulonephritis by inducing the inflammatory process. CTGF is a potentiate target for the treatment of glomerulonephritis.

## Background

Glomerulonephritis causes inflammation in glomeruli and occurs alone or as part of diseases such as vasculitis, systemic lupus erythematosus, cancer, and infections. Glomerulonephritis is a common cause of end-stage renal failure. Severe and prolonged inflammation can damage glomeruli and lead to kidney fibrosis. Kidney fibrosis is the unifying pathological feature of diverse renal disease. Emerging evidence suggests that connective tissue growth factor (CTGF) is a key player in the progression of kidney fibrosis. In addition, CTGF is known to participate in cell migration, proliferation, and inflammation. The efficacy of CTGF inhibition previously observed in a wide variety of animal models is now being evaluated in clinical trials. Therefore, CTGF appears to be a candidate therapeutic target for kidney disease. In this review, we present the current knowledge of the involvement of CTGF in kidney disease, especially glomerulonephritis.

## Connective tissue growth factor

**Fig. 1:**
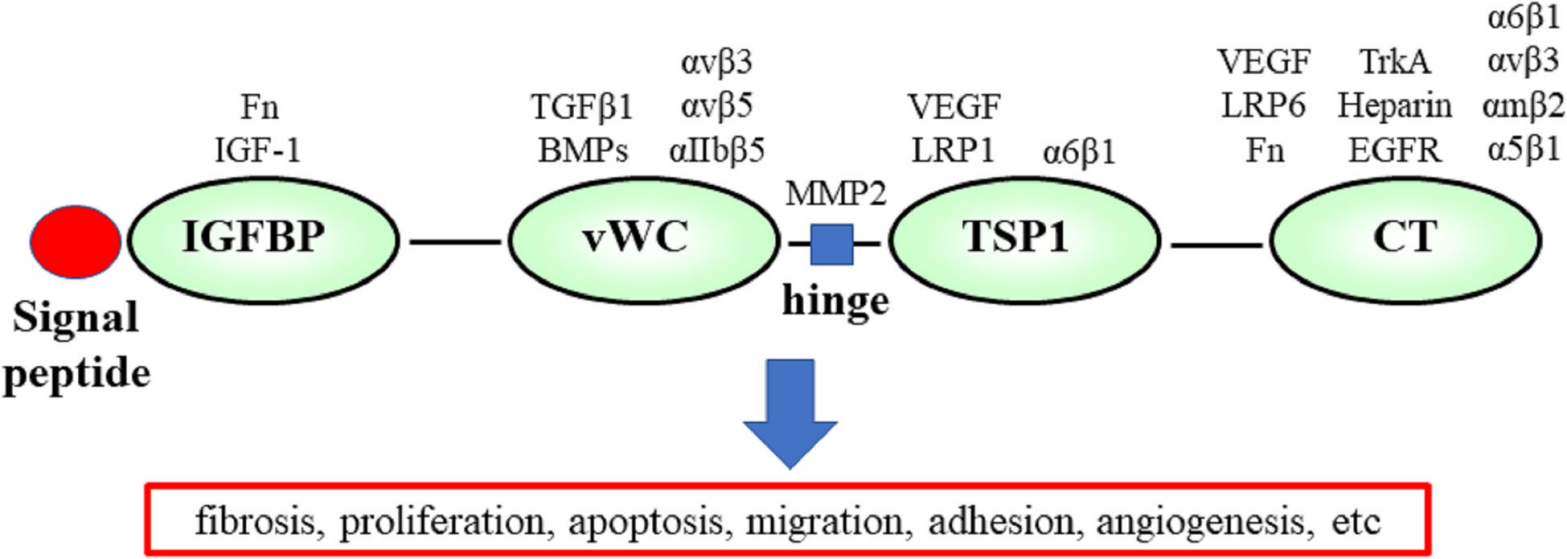
Schematic representation of the CTGF structure and interaction with the molecules. IGFBP, insulin-like growth factor binding protein domain; vWC, von Willebrand factor C domain; TSP-1, thrombospondin type 1 repeat domain; CT, C-terminal domain. Integrins were shown in each α and β subunits

CTGF/CCN2 is a member of CCN family of matricellular proteins. CTGF was isolated with an antiserum directed against the platelet growth factor from human endothelial cells in 1991. CTGF is a 36- to 38-kDa cysteine-rich secreted protein with 349 amino acids [1]. CCN family of human proteins contains six members. The name of CCN family is derived from the first letter of the first three identified members of the family CCN1-CCN3. The family members other than CTGF are cysteine-rich angiogenic inducer 61 (Cyr61/CCN1), nephroblastoma overexpressed genes (Nov/CCN3), and Wnt-inducible signaling pathway proteins 1–3 (WISP1-3/CCN4-6). CCN proteins are numbered in the order of their discovery, as proposed in 2003 [2]. Except for CCN5 which lacks domain 4, these proteins share a multimodular structure, with an N-terminal secretory signal peptide followed by four distinct conserved domains: the insulin-like growth factor-binding protein domain (domain 1; IGFBP), von Willebrand factor domain (domain 2; vWC), thrombospondin type 1 repeat (domain 3; TSP1), and a cystine knot (domain 4; CT). A hinge region susceptible to protease cleavage links domains 1 and 2, and domains 3 and 4 (Fig. 1). Human CTGF gene is located on chromosome 6q23.1 and has five exons that each encodes a signal peptide and domains 1 to 4 [3].

CTGF not only acts through their own putative receptors but also modifies various growth factors and cytokines. The specific receptor of CTGF has not been identified, and each domain of CTGF can bind to multiple ligands. These includes insulin-like growth factor-1 (IGF-1), fibronectin (domain 1: IGFBP), TGF-β1, bone morphogenetic factors, α5β3 integrin (domain 2: vWC), low-density lipoprotein receptor-related protein 1 (LRP-1), VEGF (domain 3: TSP1) and Wnt, integrins, heparan sulfate proteoglycan, LRPs, and epidermal growth factor receptor (EGFR; domain 4: CT). Thus, CTGF can simultaneously interact with several factors with their four hands. As the microenvironment differs depending on the types of cells and tissues and differentiation stages of these cells, the diverse biological actions of CTGF on various types of cells and tissues would depend on this difference in microenvironment [4].

## CTGF and development, and physiological functions

CTGF is expressed in various tissues in midgestation embryos, with the highest levels found in vascular tissues and maturing chondrocytes. Analysis of CTGF knockout mice reveals that CTGF deficiency leads to skeletal dysmorphisms due to impaired chondrocyte proliferation and extracellular matrix composition. CTGF is important for cell proliferation and matrix remodeling during chondrogenesis and is a key regulator coupling extracellular matrix remodeling to angiogenesis at the growth plate [5]. In kidney development, CTGF mRNA is presented in the immediate precursors of glomerular visceral and parietal epithelial cells in the comma- and S-shaped stages, but not in the earlier stages of nephron development. During the maturating glomerular stages, CTGF mRNA expression is maximal and present only in differentiating glomerular epithelial cells. CTGF protein is also present in the precursors of mesangial cells and glomerular endothelium [6]. The role of CTGF in kidney development cannot be excluded, but Falk et al. reported that 90% CTGF reduction does not lead to structural changes and albuminuria [7].

## CTGF and kidney fibrosis

Kidney fibrosis is a common pathological feature in chronic kidney disease and characterized by glomerulosclerosis and tubulointerstitial fibrosis. Various cytokines and growth factors are reportedly involved and associated with fibrogenic and inflammatory processes. Of these, TGF-β has been shown to play a central role in the development of renal fibrosis [8]. Igarashi et al. reported that CTGF is induced by TGF-β1 in wound healing and that there is a strong correlation between skin sclerosis and CTGF expression in the dermal fibroblasts of patients with systemic sclerosis [9, 10]. Mice overexpressing CTGF in fibroblast are susceptible to acceleration of tissue fibrosis that affects the skin, lung, kidney, and vascular system, most notably the small arteries [11]. In addition, CTGF-dependent activation of the tropomyosin-related kinase A receptor induces TIEG-1, a transcriptional receptor of Smad7, which represses Smad7, a natural receptor of TGF-β signaling. Thus, activation of CTGF increases phosph-Smad2/3, promoting transcription of Smad-responsive genes including CTGF itself. These results indicate that CTGF may be involved in fibrosis.

CTGF expression in fibrosis is also reported to occur in the kidney area. Exposure of mesangial cell to recombinant human CTGF significantly increased production of fibronectin and collagen type I. Induction of CTGF in rat mesangial cells due to high glucose levels is mediated by TGF-β [12]. The study of human kidney biopsy samples from various kidney diseases has revealed that CTGF expression level is increased in glomerulosclerosis and tubulointerstitial fibrosis [13]. Thereafter, many animal and in vitro experiments have demonstrated the pivotal role of CTGF in kidney fibrosis.

Relationship of CTGF expression levels in plasma or urine with kidney function has been reported [14, 15]. In patients with CKD, an independent association is observed between plasma CTGF level and estimated glomerular filtration rate (eGFR). In addition, plasma CTGF level correlates with residual kidney function in patients with end-stage kidney disease [14].

An interventional study of an animal model is first reported by Yokoi et al. Treatment of CTGF antisense oligonucleotide markedly attenuates the induction of fibronectin and collagen expressions in the rat unilateral ureteral obstruction (UUO) model [15]. Another study also showed the efficacy of CTGF inhibition by CTGF antisense oligonucleotide in subtotal nephrectomy of TGF-β transgenic mice [16].

In diabetes, the role of CTGF in disease development has been reported. Increased CTGF expression has been documented both in glomeruli and in tubulointerstitium [13]. Urinary CTGF is elevated as a result of both increased local production and reduced reabsorption due to tubular dysfunction and correlates with albuminuria and GFR. Thus, urinary CTGF might be as a suitable marker of diabetic nephropathy [17]. Overexpression of CTGF in the podocytes of a streptozotocin (STZ)-induced diabetes model is sufficient to exacerbate proteinuria and mesangial expansion through functional impairment and loss of podocytes [18]. In a 16-week STZ-induced diabetic nephropathy model, CTGF heterozygous mice (CTGF +/−) with 50% lower CTGF expression develop less albuminuria, mesangial expansion, and glomerular basement thickness [19]. In cultured embryonic fibroblasts from wild-type mice, glucose increases the expressions of pro-collagens 1 and 4, fibronectin, and TSP1. By contrast, activation of these genes by high glucose is attenuated in CTGF+/− embryonic fibroblasts from wild-type mice [20]. On the other hand, Falk et al. reported that a heterozygous deletion of CTGF does not prevent severe kidney fibrosis. They examined the effect of CTGF on the progression of renal scarring in long-term STZ-induced diabetic nephropathy, in the advanced stage of obstructive nephropathy following UUO and in aristolochic acid (AA)-induced tubulotoxic nephritis by using heterozygous CTGF knockout mice. Unlike in mild and relatively early STZ-induced diabetic nephropathy, scarring of severely and chronically damaged kidneys induced by STZ, UUO, and AA is not attenuated by a 50% reduction in CTGF levels relative to normal levels [21].

Possible efficacy of anti-CTGF therapy has been explored by a genetic deletion and neutralizing antibody. Of these, FG-3019, a human monoclonal antibody to CTGF, has been used in some animal models, including pulmonary fibrosis, peritoneal fibrosis, and systemic sclerosis. These studies showed successful treatment for fibrosis by inhibition of CTGF [22]. In addition, FG-3019 has also humper tumor growth in mouse models of pancreatic cancer, ovarian cancer, and melanoma [23, 24]. Moreover, FG-3019 has been used in clinical trials for pulmonary fibrosis and pancreatic cancer and no serious adverse effects have been observed [25]. Although treatment for diabetic kidney disease with microalbuminuria using FG-3019 is well tolerated and associated with decreased albuminuria, there are no active trials in renal field [26].

## CTGF and glomerulonephritis

Acute and chronic inflammation usually precedes the development of organ fibrosis. Activated inflammatory cells release many factors, including profibrotic cytokines such as TGF-β, and chronic inflammation leads to the development of fibrosis. CTGF is well known to participate in this fibrotic process. Apart from this fibrotic effect, several reports have showed the upregulation of CTGF expression in glomerulonephritis. Glomerulonephritis often develops from intra-glomerular activation via the classical or alternative complement pathway. Immune complexes can form different compartment of the glomerulus, which determines the resulting histopathological lesion, as different glomerular cell types are primarily activated. The result of histological lesions determined the classification of glomerulonephritis. Immune complex deposition in mesangial cell activates mesangial cells lead to mesangioproliferative glomerulonephritis, such as IgA nephropathy. Subendothelial immune complex deposition activates endothelial cells, as seen in lupus nephritis classes III and IV. Subepithelial immune complex deposition activate podocytes, as seen in membranous nephropathy usually cause massive proteinuria. Immune complex deposition in glomerular basement membrane (GBM) induces anti-GBM disease. Anti-neutrophil cytoplasmic antibody (ANCA)-associated glomerulonephritis develops the absence of immune complex deposits, as it is driven by both ANCA and cellular immunity [27]. Ito et al. showed that CTGF is strongly upregulated in the extra-capillary and severe mesangial proliferative lesions of IgA nephritis, crescentic glomerulonephritis, and focal segmental sclerosis in various human kidney biopsy samples [6, 13]. Another study reported that CTGF is strongly expressed in cellular and fibrocellular crescents and proposed that it is involved in extracellular matrix production by parietal epithelial cells [28]. The mRNA expression of CTGF in kidney biopsy samples from chronic glomerulonephritis is higher than that in control samples [29].

Animal models of glomerulonephritis also reported increased expression of CTGF. In anti-Thy-1.1 nephritis, CTGF mRNA expression is strongly increased in mesangial proliferative and extra-capillary lesions. Glomerular CTGF expression is maximal on day 7, in association with increased TGF-β1 mRNA and protein expression levels. The kinetics of CTGF expression strongly suggests a role in glomerular repair, possibly downstream of TGF-β, in this model of transient renal injury [30]. In the acute phase of rat crescentic glomerulonephritis, a major component of crescents was macrophages, which do not express CTGF mRNA. However, in the advanced phase, crescentic cells strongly express CTGF mRNA and epithelial marker but do not express the macrophage marker ED1, which suggests that parietal epithelial cells synthesize CTGF. Blockade of endogenous CTGF using antisense oligonucleotide significantly attenuates TGF-β1 and PDGF-BB-induced extracellular matrix accumulation in parietal epithelial glomerular cells [28].

The relationship of plasma and urine CTGF levels with kidney function in glomerulonephritis was previously reported. CTGF mRNA is expressed at the site of chronic tubulointerstitial damage and correlated with the degree of damage [13]. In patients with anti-neutrophil cytoplasmic antibodies-associated glomerulonephritis, plasma CTGF levels are associated with cellular crescents but are not correlated with renal function. The plasma CTGF level at baseline predicted renal survival more accurately than the acute glomerular nephritis classification [31]. In lupus nephritis, renal CTGF mRNA expression correlates inversely with baseline GFR and was also higher in patients with subsequent decline in GFR [32]. These results indicate the relationship of CTGF with glomerulonephritis.

Anti-GBM nephritis is an animal model commonly used to study a type of immune complex-mediated glomerulonephritis [33]. Anti-GBM nephritis is caused by autoantibodies specific for α3 chain of type IV collagen. Neutrophil recruitment to the kidney starts several hours after the induction of anti-GBM nephritis and its mediated by interleukin-17A (IL-17)-producing γδT cell. The adaptive immune response is initiated by mature dendritic cells that depend on CC-chemokine receptor 2 (CCR2). In earlier stage, immune responses that are mediated by Th17 cells which recruit neutrophils and macrophages cause sustained kidney inflammation [27]. Usually, CTGF is known to be a downstream mediator of TGF-β. Blockade of TGF-β in the early stage of anti-GBM nephritis in rat ameliorates renal function and histological changes such as crescentic formation and interstitial fibrosis [34]. The gene expression profile of anti-GBM glomerulonephritis revealed that CTGF is expressed as early as on the first day of disease induction preceding TGF-β1 expression [35]. Rodrigues-Diez et al. showed that the C-terminal domain 4 of CTGF induced renal Th17 inflammatory response. In vitro, stimulation of human CD4^+^ T lymphocytes with CTGF domain 4 results in differentiation of the Th17 phenotype [36]. These results mean that CTGF might be involved in inflammatory responses and is a candidate for therapeutic target for glomerulonephritis.

**Fig. 2:**
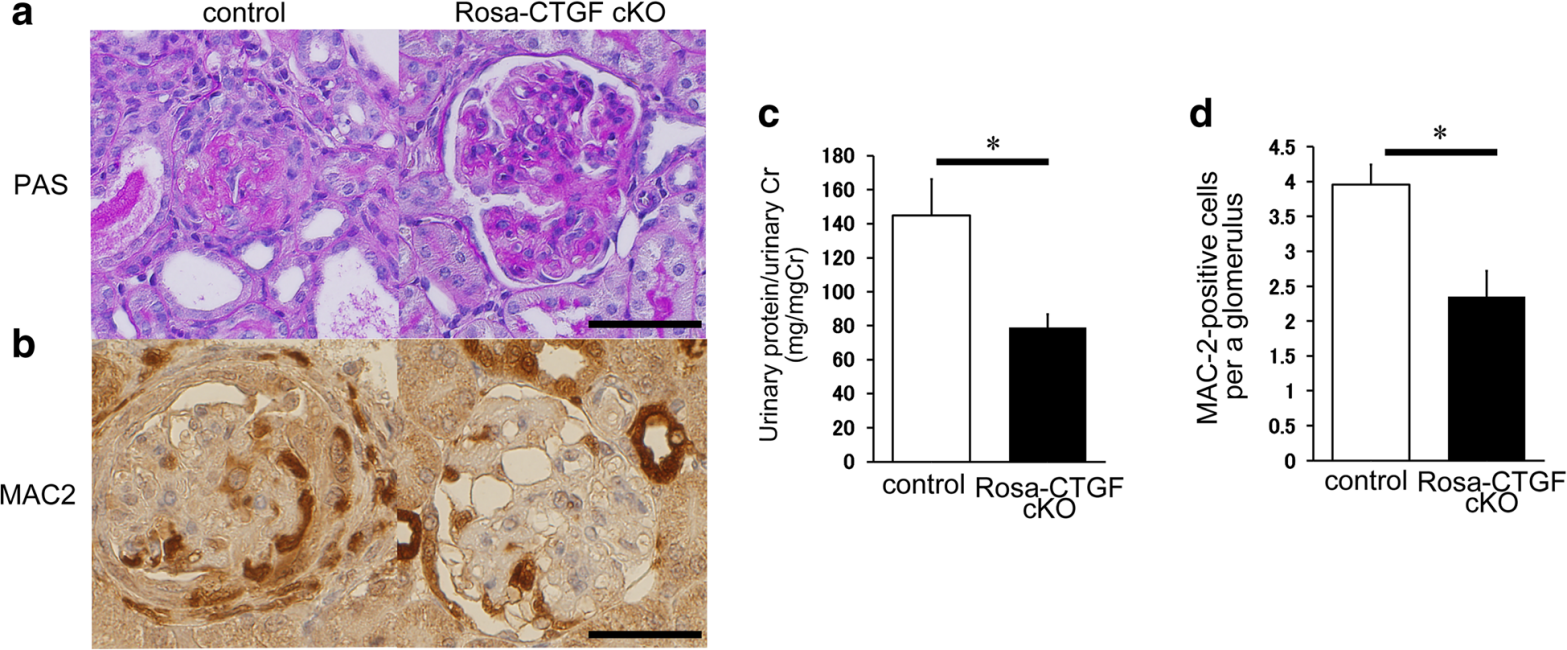
Macrophage recruitment in Rosa-CTGF cKO mice with anti-GBM nephritis at the earlier stage. **a** Representative photomicrographs of the kidneys at 1 week after induction of anti-GBM nephritis (PAS staining). Left upper panel, control mice with anti-GBM nephritis; right upper panel show, Rosa-CTGF cKO mice with anti-GBM nephritis. Bar represents 50 μm. **b** Immunohistochemical studies for MAC-2 at 1 week after induction of anti-GBM nephritis. Bar represents 50 μm. **c** Changes in proteinuria at 1 week after induction of anti-GBM nephritis. **d** The number of MAC-2-positive cells at 1 week after induction of anti-GBM nephritis. Values are expressed as means ± s.e. *P < 0.05, **P < 0.01 vs. control GBM

Complete deletion of CTGF is a desired in an experimental approach for evaluating the contribution of CTGF to the development of renal disease. However, CTGF knockout mice die shortly after birth. To investigate the role of CTGF in the glomerulonephritis model and the contribution of endogenous CTGF expression, we generated a full length of CTGF floxed mice and established tamoxifen-inducible systemic CTGF knockout (Rosa-CTGF cKO) mice by crossing Rosa-CreER^T2^ mice [37]. The gene expression of CTGF in the kidneys of Rosa-CTGF cKO mice is decreased by 80%. After induction of anti-GBM nephritis, Rosa-CTGF cKO mice exhibit 50% reduction of proteinuria and decreased crescent formation and mesangial expansion as compared with the control mice. In addition to the increases in the expression levels of fibrotic makers such as *Tgfβ1*, *Acta2*, and *Fn1*, the glomerular mRNA expression of MCP-1 (*Ccl2*) and F4/80 (*Adgre1*) is increased in the control mice with anti-GBM nephritis, and this increase is reduced in the Rosa-CTGF cKO mice with nephritis. Accumulation of MAC2-positive cells in glomeruli is also reduced in Rosa-CTGF cKO mice. It is interesting that this amelioration of anti-GBM nephritis is not observed in podocyte-specific CTGF deletion. Furthermore, mesangial cell CTGF cKO mice with nephritis show similar phenotype to Rosa-CTGF cKO mice [38]. In addition, Rosa-CTGF cKO mice with peritoneal fibrosis also exhibit almost 50% reduction in MAC-2 (macrophage marker)-positive cell infiltration and *Cd68* mRNA expression in the peritoneum (Fig. 2) [39]. These results suggest that CTGF from mesangial cell, not podocytes, may be required for the upregulation of MCP-1expression not only in anti-GBM nephritis but also in other types of glomerulonephritis, such as IgA nephropathy, because CTGF expression and accumulation of macrophages in the mesangial area are documented in these glomerular diseases [38].

## Role of CTGF in adhesion and migration

During development of inflammation, transmigration of leukocytes to the inflammatory site is a major step. Inflammatory stimuli activate endothelial cells to express adhesion molecules and chemokines which recruit leukocytes. An increasing number of studies have shown the function of CTGF in adhesion and migration.

CTGF modulates the expression of inflammatory mediators, including cytokines and chemokines through distinct signaling pathways in various cell systems [40]. Direct application of CTGF osteoarthritis synovial fibroblast increases the MCP-1 expression in a time- and dose-dependent manner. CTGF-mediated MCP-1 production is attenuated by α_V_β1 integrin-neutralized antibody. Pretreatment with focal adhesion kinase (FAK), MEK, AP-1, and NF-κB inhibitor also inhibits the potentiating action of CTGF. CTGF-mediated increase in NF-κB and AP-1 luciferase activities are inhibited by FAK, MEK, and ERK inhibitors [41]. In vivo, Sanchez-Lopes reported that systemic administration of CTGF in mice for 24 h induces marked infiltration of T lymphocytes and macrophages in the renal interstitium and leads to elevated renal NF-κB activity. Administration of CTGF increases the renal expression of chemokines (MCP-1 and RANTES) and cytokines (INF-ϒ and IL-6) that recruit immune cells and promote inflammation [42]. In rat mesangial cells, CTGF expression induces production of fractalkine, MCP-1, and RANTES in a time- and dose-dependent manner via the p42/44 MAPK-, PI3-K/AKT-, and NF-κB-dependent signal pathways [43]. MCP-1 expression is reduced by CTGF inhibition in TGF-β1-treated mesangial cells. Treatment with recombinant CTGF can overcome this effect of endogenous CTGF inhibition. In tubule-epithelial cells, CTGF increases MCP-1 gene expression through activation of NF-κB and mitogen-activated protein kinase [42]. Thus, CTGF is thought to regulate proinflammatory cytokines and chemokines and induces leukocyte migration in kidney inflammation.

**Fig. 3:**
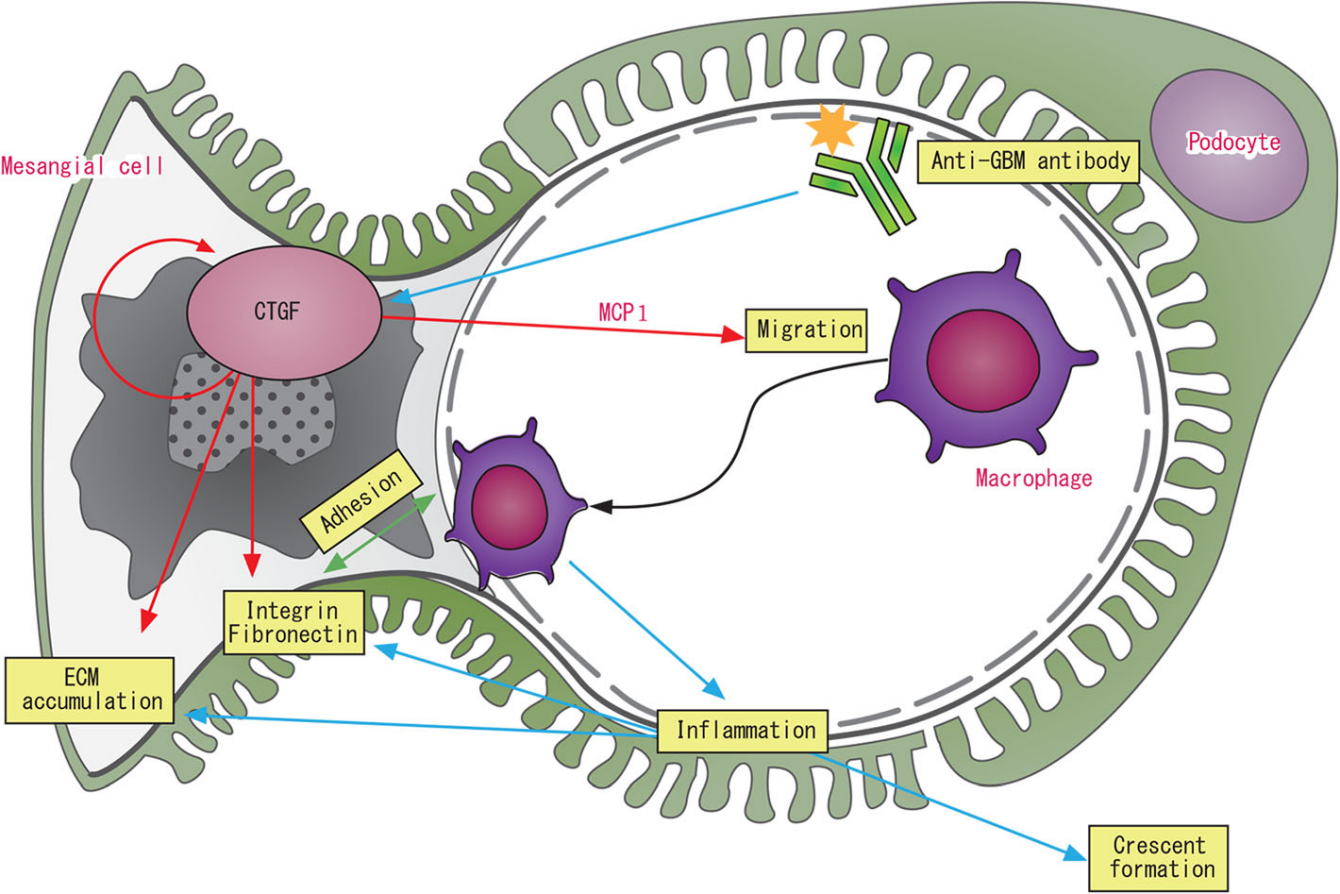
CTGF mediates chemotaxis and adhesion of macrophages as well as ECM production in mesangial cells. Anti-GBM nephritis elicits upregulation of CTGF in mesangial cells. CTGF derived from mesangial cells increases MCP-1 (CCL2) expression, which induces macrophage migration and ECM proteins, including integrin αv and fibronectin, which contribute macrophage adhesion with mesangial cells

Previous reports have demonstrated that CTGF enhances adhesion through interactions with integrins and fibronectin in various cell types. These results showed that the absence of CTGF prevents cell adhesion and treatment of CTGF increases cell adhesion. This CTGF-mediated adhesion occurs through integrin and fibronectin expressions [44]. As regards macrophage or monocyte adhesion, Schober et al. reported that activated monocytes adhere to Cyr61 (CCN1) and CTGF through α_M_β2 integrin and cell surface heparan sulfate proteoglycans [45]. Another report showed that CTGF induces peripheral blood mononuclear cell (PBMC) migration in a dose-dependent manner. In the presence of heparin, which binds to CTGF, the chemotactic response to CTGF is reduced. Cell surface heparin sulfate is required for CTGF-mediated PBMC migration [46]. Osteoarthritis synovial fluid and supernatants from CTGF-treated osteoarthritis synovial fibroblasts increase migration of monocytes. In addition, CTGF-mediated migration is inhibited by MEK and ERK inhibitors [41]. Mesangial cell adhesion and CTGF are also reported. CTGF significantly increases cell surface α5β1 integrin levels relative to the basal levels in human mesangial cells (HMC). CTGF and TGF-β increased cell adhesion to fibronectin, the main α5β1 substrate. Antisense CTGF reduces the number of adherent cells with TGF-β stimulation. CTGF controls α5β1 expression by HMC in vitro [47]. We investigated the effects of CTGF on the adhesion of macrophages to activated mesangial cells. Fluorescein-dye-labeled RAW264.7 cells are co-cultured with recombinant TNF-α-stimulated mesangial cells on culture plates. The increase in macrophage adhesion by TNF-α stimulation is significantly inhibited by CTGF knockdown in mesangial cells, and this reduction is negated by exogenous CTGF administration. These results suggest that CTGF induces macrophage accumulation in glomerulonephritis by enhancing both chemotaxis and adhesion and that reduction of CTGF expression, particularly in mesangial cells, ameliorates nephritis via inhibition of macrophage infiltration (Fig. 3) [38].

## CTGF and inflammatory mediator

The regulation of CTGF expression by an inflammatory mediator has been reported. It was found that the effect of TNF-α on CTGF expression is dependent on cell systems or exposure time. The sequences between − 244 and − 166 of the CTGF promoter were necessary for TNF-α to modulate CTGF expression [48]. TGF-β1 induces CTGF gene expression via Smad-binding element (SBE) and a unique TGF-β1 response element which is located between − 162 and − 128 of the CTGF promoter [49]. Short-term treatment of mesangial cells with TNF-α, like with TGF-β, significantly increases secreted CTGF per cell. TNF-α combined with TGF-β further increases CTGF secretion and mRNA levels and reduces proliferation. However, long-term treatment with TNF-α or TGF-β alone does not increase CTGF protein levels [50]. In synovial cells, TNF-α can also induce CTGF production [51]. By contrast, TNF-α downregulated CTGF in human lung endothelial cells and in normal and scleroderma fibroblasts in a dose- and time-dependent manner [52, 53].

Several reports indicated that CTGF modulates the expression of inflammatory mediators. Stimulation with CTGF induces TNF-α expression in macrophage [38]. Osteoarthritis synovial fibroblast stimulation with CTGF induces concentration-dependent increases in IL-6 expression level. CTGF-mediated IL-6 production is attenuated by αvβ5 integrin-neutralized antibody [54]. In tubule-epithelial cells, CTGF increases the IL-6 gene expression through activation of NF-κB and mitogen-activated protein kinase [42]. In clinical, the serum level of CTGF in rheumatoid arthritis (RA) was higher than in normal controls and active RA patients showed higher serum CTGF level than inactive RA patients. Furthermore, CTGF level was decreased by infliximab, anti-TNF-α antibody [55]. These results suggest that CTGF induces inflammatory mediators.

## Conclusions

CTGF is a downstream mediator of the profibrotic properties of TGF-β. In addition to fibrosis, CTGF has multiple functions, including cell adhesion and migration. CTGF expression is upregulated in glomerulonephritis. Deletion of CTGF can ameliorate anti-GBM glomerulonephritis by reducing macrophage accumulation in mice. Further studies are required to investigate the use of CTGF as a potential target for the treatment of glomerulonephritis.

## Abbreviations

CTGF: Connective tissue growth factor
GBM: Glomerular basement membrane
GFR: Glomerular filtration rate
IFN-γ: Interferon-gamma
IL-6: Interleukin-6
MCP-1: Monocyte chemoattractant protein-1
RANTES: Regulation and activation, normal T cell expressed and secreted
STZ: Streptozotocin
TGF-β1: Transforming growth factor beta1
TNF-α: Tumor necrosis factor alpha
VEGF: Vascular endothelial growth factor
